# Autoantibodies to transcription intermediary factor (TIF)1β associated with dermatomyositis

**DOI:** 10.1186/ar3802

**Published:** 2012-04-18

**Authors:** Minoru Satoh, Jason YF Chan, Steven J Ross, Yi Li, Yoshioki Yamasaki, Hidehiro Yamada, Monica Vazquez-del Mercado, Marcelo H Petri, Luis J Jara, Miguel A Saavedra, Claudia Cruz-Reyes, Eric S Sobel, Westley H Reeves, Angela Ceribelli, Edward KL Chan

**Affiliations:** 1 Division of Rheumatology and Clinical Immunology, Department of Medicine, University of Florida, P.O.Box 100221, 1600 SW Archer Rd, Gainesville, FL 32610-0221, USA; 2 Department of Pathology, Immunology, and Laboratory Medicine, University of Florida, P.O.Box 100221, 1600 SW Archer Rd, Gainesville, FL 32610-0221, USA; 3 Department of Oral Biology, University of Florida, P.O.Box 100424, 1600 SW Archer Rd, Gainesville, FL 32610-0424, USA; 4 Division of Rheumatology, Department of Internal Medicine, St. Marianna University School of Medicine, 2-16-1 Sugao, Miyamae-ku, Kawasaki, Kanagawa, 216-8511, Japan; 5 Departamento de Biología Molecular y Genómica, Instituto de Investigación en Reumatología y del Sistema Músculo Esquelético, Centro Universitario de Ciencias de la Salud, Universidad de Guadalajara, Sierra Mojada 950, Guadalajara, Jalisco, CP 44340, México; 6 División de Medicina Interna, Departamento de Reumatología, Hospital Civil 'Dr. Juan I. Menchaca', Salvador de Quevedo y Zubieta N° 750, CP 44340, Guadalajara, Jalisco, México; 7 Direction of Education and Research, Hospital de Especialidades 'Dr. Antonio Fraga Mouret', Centro Médico Nacional 'La Raza', IMSS, Seris/Zaachila s/n, Colonia La Raza, Delegación Azcapotzalco, CP 02990, Mexico City, México; 8 Universidad Nacional Autónoma de México, Avenida Universidad 3000, Delegación Coyoacán, CP 04510, Mexico City, México; 9 Department of Rheumatology, Hospital de Especialidades 'Dr. Antonio Fraga Mouret', Centro Médico Nacional 'La Raza', IMSS, Seris/Zaachila s/n, Colonia La Raza, CP 02990, Mexico City, México

## Abstract

**Introduction:**

Myositis specific autoantibodies are associated with unique clinical subsets and are useful biomarkers in polymyositis/dermatomyositis (PM/DM). A 120 kD protein recognized by certain patients with DM was identified and clinical features of patients with this specificity were characterized.

**Methods:**

The 120 kD protein recognized by a prototype serum was purified and identified by mass spectrometry and immunological methods. Autoantibody to this 120 kD protein was screened in sera from 2,356 patients with various diagnoses from four countries, including 254 PM/DM, by immunoprecipitation of ^35^S-methionine labeled K562 cell extracts. Clinical information of patients with this specificity was collected.

**Results:**

The 120 kD protein, which exactly comigrated with PL-12, was identified as transcription intermediary factor TIF1β (TRIM28) by mass spectrometry and validated by immunoassays. By immunofluorescence, anti-TIF1β positivity showed a fine-speckled nuclear staining pattern. Four cases of anti-TIF1β were identified; all are women, one each in a Japanese, African American, Caucasian, and Mexican individual. Three had a diagnosis of DM and one case was classified as having an undifferentiated connective tissue disease with an elevated CPK but without significant muscle symptoms. This individual also had a history of colon cancer, cervical squamous metaplasia and fibroid tumors of the uterus. Myopathy was mild in all cases and resolved without treatment in one case. The anti-TIF1β specificity was not found in other conditions.

**Conclusions:**

Anti-TIF1β is a new DM autoantibody associated with a mild form of myopathy. Whether it has an association with malignancy, as in the case of anti-TIF1γ, or other unique features will need to be evaluated in future studies.

## Introduction

Autoantibodies to cellular constituents are clinically important biomarkers associated with particular diagnoses, specific clinical features or subsets of disease, helping to establish a diagnosis, and/or predicting organ involvement and prognosis [1,2]. In particular, in polymyositis/dermatomyositis (PM/DM) and scleroderma (systemic sclerosis, SSc) patients can be classified into several subsets associated with characteristic clinical features based on specific autoantibodies, since coexistence of other disease-specific autoantibodies is uncommon [3]. Each myositis specific antibody (MSA) is associated with a unique clinical subset. For example, the anti-synthetase syndrome was named for the presence of anti-Jo-1 and other autoantibodies to aminoacyl tRNA synthetases found in a subset of patients with PM/DM whose clinical presentation was dominated by interstitial lung disease (ILD), Raynaud's phenomenon, arthritis, fever, and mechanic's hands [3,4]. Although new autoantibody specificities have been reported, approximately 40% to 50% of patients with PM/DM are still without a known MSA compared with only approximately 15% in SSc without association to known SSc antibodies [2]. Thus, identifying new MSA may help in monitoring PM/DM patients and several new clinically significant autoantibodies associated with DM including anti-p155/140 [5-11], anti-CADM (clinically amyopathic DM) 140/MDA5 (melanoma differentiation associated antigen 5) [10,12-14], anti-SAE (small ubiquitin-like molecule activating enzyme) and anti-MJ/NXP-2 have been reported recently [15,16]. Among these, anti-p155/140 has been studied extensively in a very short period of time due to its strong association with malignancy [5-9,11] which was confirmed by a recent meta-analysis [17]. However, this association does not appear to apply to children [7] or young adults [11]. p155 was identified as transcription intermediary factor1γ, (TIF1γ, also known as tripartite motif (TRIM) 33) [18]. A recent study in Japanese patients has identified the p140 as TIF1α and another related molecule TIF1β has also been identified as a target of autoantibodies in DM [11]. In the present study, we have independently identified the approximately 120 kD autoantigenic protein as TIF1β by mass spectrometry. The presence of anti-TIF1β and clinical features of American, Mexican, and Japanese patients with this specificity were characterized.

## Materials and methods

### Patients

A total of 2,356 sera, including 1,966 subjects enrolled in the University of Florida Center for Autoimmune Diseases (UFCAD) registry from 2000 to 2010, were studied. Diagnoses of the UFCAD patients include 434 systemic lupus erythematosus (SLE), 86 PM/DM (51 PM including 12 PM-SSc overlap, 35 DM), 121 SSc, and 122 rheumatoid arthritis (RA). Additionally, sera from 36 PM/DM (13 PM, 20 DM, 3 amyopathic DM) from St. Marianna University Hospital (Kawasaki, Japan), 74 PM/DM (18 PM, 56 DM) sera from Guadalajara and Mexico City (Mexico), 58 PM/DM (25 PM, 27 DM, 6 overlap: 4 PM-SSc, 1 DM-SLE, 1 PM-RA), 57 SSc, and 113 SLE, and 52 primary anti-phospholipid syndrome (PAPS) from Spedali Civili di Brescia (Brescia, Italy) were also screened. Diagnosis of PM/DM is by physician's assessment based on Bohan's criteria (PM/DM). Other diagnoses were established by the American College of Rheumatology (ACR) (SLE, SSc, RA) or European criteria (Sjögren's syndrome). Clinical information was from database and medical records. The protocol was approved by the Institutional Review Board (IRB). This study meets and is in compliance with all ethical standards in medicine, and informed consent was obtained from all patients according to the Declaration of Helsinki.

### Materials and methods

#### Immunoprecipitation

Autoantibodies in sera were screened by immunoprecipitation using ^35^S-methionine labeled K562 cell extracts [19]. Specificity of autoantibodies was determined using previously described reference sera. Analysis of RNA components of autoantigens was by urea-PAGE and silver staining (Silver Stain Plus, Bio-Rad, Hercules, CA, USA) [20].

#### Identification of proteins by LC-MS/MS

The new unidentified autoantigen of 120 kD protein was purified using serum from the prototype patient. Immunoglobulin G (IgG) from 20 μl of serum was crosslinked to protein A Sepharose beads using dimethylpimerimidate. Protein was then affinity-purified from a cell extract of 4 × 10^8 ^K562 cells and fractionated by SDS-PAGE. The 120 kD protein band was cut following silver staining of the gel, trypsin-digested and analyzed by liquid chromatography - tandem mass spectrometry (LC-MS/MS) analysis on a hybrid quadrupole-TOF mass spectrometer (QSTAR elite, Applied Biosystems) at UF ICBR Protein Core. Tandem mass spectra were extracted by ABI Analyst version 2.0. All MS/MS samples were analyzed using Mascot (Matrix Science, London, UK; version 2.2.2). Scaffold (version Scaffold-02-03-01, Proteome Software Inc.) was used to validate MS/MS based peptide (> 95.0% probability) and protein identifications (> 99.0% probability and at least two identified unique peptides).

#### Affinity purification of the TIF1β and western blot

Identity of the approximately 120 kD protein as TIF1β was verified by immunoprecipitation followed by western blot (IP-WB) using an extract from 5 × 10^6 ^K562 cells and 2 μl of human serum. Purified proteins were fractionated in 8% acrylamide SDS-PAGE, transferred to nitrocellulose filter and probed with mouse anti-TIF1β monoclonal antibodies (mAb) (EMD Millipore, Billerica, MA, USA), followed by horseradish peroxidase-conjugated (HRP) goat anti-mouse Ig light-chain antibodies (Jackson ImmunoResearch Laboratories, Inc. West Grove, PA, USA) and developed with SuperSignal West Femto Chemiluminescent Substrate (Thermo Scientific, Rockford, IL, USA).

In another experiment, TIF1β was affinity purified from an extract of 2 × 10^8 ^K562 cells using 10 μg of anti-TIF1β mAb, and purified proteins were fractionated by SDS-PAGE and transferred to nitrocellulose filter. Strips (2 mm width) of nitrocellulose filter were probed with mouse mAb and human autoimmune sera. Strips incubated with mouse mAb were then incubated with HRP goat anti-mouse Ig light-chain antibodies and developed, while samples probed with human sera were incubated with HRP-donkey IgG F(ab)'2 anti-human IgG (γ-chain specific) antibodies (Jackson ImmunoResearch Laboratories, Inc.) and developed.

#### Immunofluorescent antinuclear antibodies

Immunofluorescent antinuclear/cytoplasmic antibodies (HEp-2 ANA slides; INOVA Diagnostics, San Diego, CA, USA) were tested using a 1:80-diluted human serum or 2 μg/ml mouse mAb to TIF1β. Secondary antibodies were DyLight 488 donkey IgG F(ab)'2 anti-human or -mouse IgG (1:200 dilution, γ-chain-specific, Jackson ImmunoResearch Laboratories).

#### ELISA

Antigen-capture ELISA for anti-TIF1β was performed using 2 μg/ml mouse mAb to TIF1β (Millipore), following a protocol that was used for other autoantibody systems [21]. Antibodies to TIF1α (p140 of p155/140), TIF1β, and TIF1γ (p155) were also tested using full-length recombinant proteins from Abnova (TIF1α and β, Taipei, Taiwan) and OriGene Technologies (TIF1γ, Rockville, MD, USA), respectively. Wells of microtiter plates were coated using 0.5 μg/ml of protein and ELISA was performed following standard protocol as described [22].

## Results

First, the 120 kD protein was affinity purified using the human prototype serum and the identity of the protein was determined by LC-MS/MS. Sixteen unique (total of 18) peptides that contained sequences identical to TIF1β were isolated as follows: aa128-136 DIVENYFMR (*n *= 2), aa254-261 KLLASLVK, aa283-290 QVSDVQKR, aa297-304 MAILQIMK, aa310-319 GRVLVNDAQK, aa312-319 VLVNDAQK, aa312-327 VLVNDAQKVTEGQQER, aa331-337 QHWTMTK, aa408-427 IVAERPGTNSTGPAPMAPPR, aa473-483 SGEGEVSGLMR (*n *= 2), aa493-507 LDLDLTADSQPPVFK, aa508-524 VFPGSTTEDYNLIVIER, aa751-767 LSPPYSSPQEFAQDVGR, aa775-790 LTEDKADVQSIIGLQR, aa 780-790 ADVQSIIGLQR, and aa796-804 MNEAFGDTK. These peptides covered 19.5% (163/835 amino acids) of the sequence of TIF1β. Since the reported molecular weight of TIF1β is approximately 120 kD, it was considered a good candidate and its identity was validated using mAb to TIF1β.

### Immunoprecipitation

**Figure 1:**
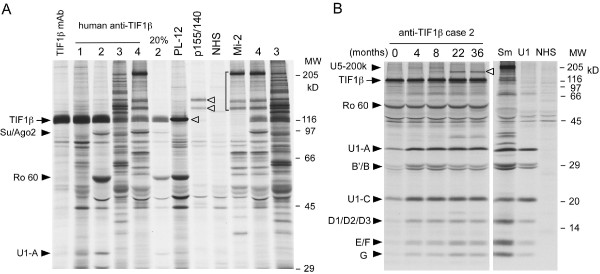
**Immunoprecipitation using ^35^S-methionine labeled K562 cell extract**. **A**. 8% SDS-PAGE. ^35^S-methionine labeled K562 cell extract was immunoprecipitated as follows: Anti-TIF1β mAb; lanes 1 to 4, anti-TIF1β positive human sera; 20% 2, 20% loading of prototype serum in lane 2; PL-12, p155/140, NHS, normal human serum; Mi-2, reference serum for each specificity. Positions of TIF1β, Su/Ago2, Ro 60, U1snRNP A (U1-A), and molecular weight markers are shown on the left. White arrowheads indicate PL-12 (lane PL-12) and p155 (TIF1γ) and p140 (TIF1α) (lane p155/140). **B**. 12.5% SDS-PAGE. Sequential sera from case two were tested by immunoprecipitation. Positions of components of UsnRNPs, TIF1β, and Ro-60 and molecular weight markers are shown. NHS, normal human serum; Sm, U1, anti-Sm and anti-U1RNP reference serum, respectively; TIF, transcription intermediary factor.

The 120 kD proteins immunoprecipitated by four sera (Figure 1A, lanes 1-4), both in size and appearance, appeared identical to the TIF1β protein immunoprecipitated by the mAb, consistent with the identity of the 120 kD proteins as TIF1β. TIF1β showed a migration pattern similar to the known myositis autoantigen PL-12 but had a broader band in contrast to the sharp band of PL-12 (Figure 1A). Two sera (lanes 1-2) had strong reactivity, while the other two (lanes 3-4) were weak. When 20% of the sample of case two was run (lane 2), it showed an appearance that was very similar to the weak ones in lanes 3-4, suggesting that they are the same proteins. Although TIF1β/TRIM28 and TIF1γ/TRIM33 are in the same family of related proteins and their interactions have been reported [23], anti-TIF1β mAb did not immunoprecipitate TIF1γ/p155 (Figure 1A, lane TIF1β mAb). Also, anti-TIF1γ (p155/140) positive serum did not immunoprecipitate TIF1β (Figure 1A, lane p155/140), consistent with a lack of crossreactivity and interactions between TIF1β and TIF1γ under the conditions used for immunoprecipitation. None of these four human sera with anti-TIF1β antibodies clearly immunoprecipitated TIF1γ/α (p155/140) (Figure 1A). However, the serum from case three was positive for anti-TIF1α by ELISA using recombinant protein (Table 1). Case four was negative for antibodies to ecombinant TIF1γ and α by ELISA. Also, none of the 23 anti-p155/140 positive sera in our cohort immunoprecipitated TIF1β (data not shown). Case four had coexisting anti-Mi-2 antibodies (Figure 1A, lane Mi-2), cases two and four had anti-Su/Ago2, and case two also had anti-Ro and U1RNP. Sequential serum samples available for case two were tested by immunoprecipitation (Figure 1B). The levels of anti-U1RNP antibodies that were weakly positive at the initial visit increased followed by the development of additional autoantibodies to an unidentified protein of approximately 160 kD (white arrowhead).

**Table 1:** Clinical features of patients with anti-TIF1β autoantibodies.

	1	2	3	4
Diagnosis	DM	UCTD	DM	DM
Symmetrical muscle weakness	P	N	P	Y
Muscle biopsy	Y	ND	ND	NA
Elevated muscle enzyme	Y	Y	Y	Y
EMG	myopathic pattern	myopathic pattern	myopathic pattern	NA
Dermatologic features	Y (G)	N	P (S)	Y (G, H, S)
Malignancy	N	Y	N	N
Interstitial lung disease	N	N	N	N
Dysphagia	Y	N	N	N
Raynaud's phenomenon	N	N	N	N
Arthritis	N	Y	N	N
CPK (U/L) initial /after Tx (lowest)	654 167	341 239	314	2414
Initial Tx	none	PSL 10 mg	PSL 40 mg	PSL 50 mg
		HCQ 200 mg NSAIDs	HCQ 200 mg	MTX 20 mg/w HCQ 150 mg
Response to Tx	NA	good	good	good
Other autoantibodies		Ro, Su, U1RNP (Sm)		Mi-2, Su
ELISA (RP) TIF1α	-	-	+	-
TIF1β	+	+	-	-
TIF1γ	-	-	-	-
antigen-capture ELISA				
TIF1α	-	-	-	-
TIF1β	+	+	weak +	weak +

DM, dermatomyositis; ELISA, enzyme-linked immunosorbent assay; EMG, electromyogram; G, Gottron; H, heliotrope; HCQ, hydroxychloroquine; MTX, methotrexate; N, no; NA, not available; ND, not done; NSAIDs, non-steroid anti-inflammatory drugs; P, possible; PSL, prednisolone; RP, recombinant protein; S, shawl sign; TIF, transcription intermediary factor; Tx, treatment; UCTD, undifferentiated connective tissue disease; Y, yes;

Since many autoantigens in PM/DM are RNA-protein complexes, RNA components in immunoprecipitates were also analyzed by silver staining. No common RNA component was detected, suggesting TIF1β is not complexed with specific RNA (data not shown).

### Confirmation of the 120 kD protein as TIF1β

**Figure 2:**
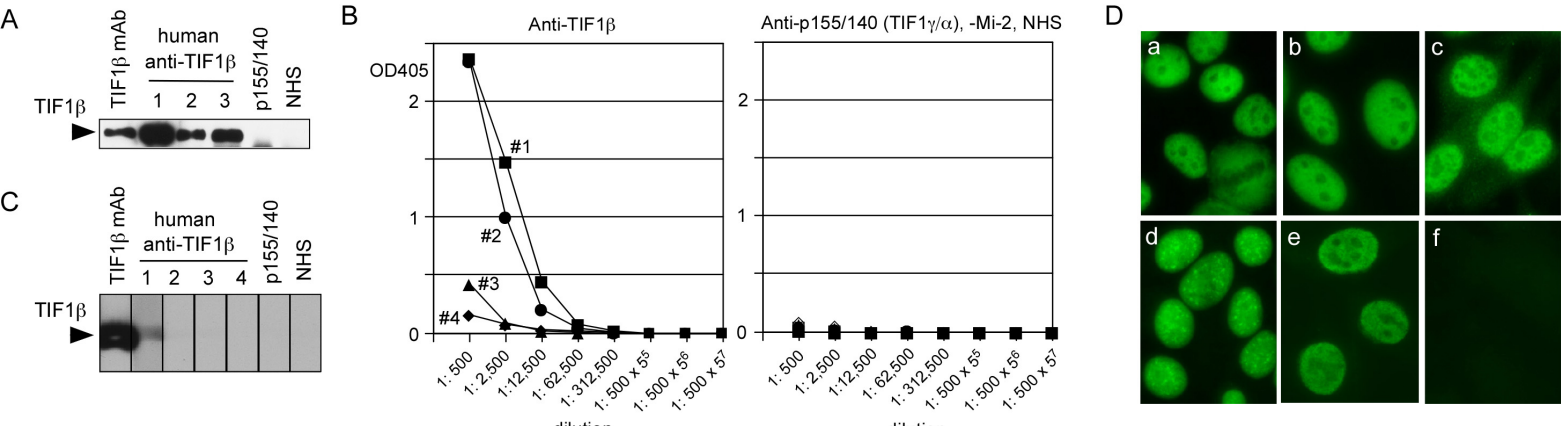
**Characterization of human anti-TIF1β positive sera**. **A**. IP-western blot. Extract from 5 × 10^6 ^K562 cells was immunoprecipitated by mouse anti-TIF1β mAb, human anti-TIF1β positive sera (lanes 1 to 3 correspond to case 1 to 3, case 3 is a weakly positive sample), human anti-p155/140 (TIF1-γ/α) positive serum, or normal human serum (NHS). Purified proteins were fractionated by 8% SDS-PAGE, transferred to nitrocellulose filter and probed with mouse anti-TIF1β mAb. Only 1/10 amount of immunoprecipitates was loaded for mAb, case 1, and 2 in order to obtain more comparable signals to case 3. **B**. Anti-TIF1β antigen-capture ELISA. Four human anti-TIF1β positive sera (left) and controls (right, 3 anti-TIF1-γ, 3 anti-Mi-2, and 2 normal human sera, NHS) were serially diluted from 1:500 and tested by antigen-capture ELISA. **C**. Western blot. **D**. Immunofluorescence staining of HEp-2 cells. HEp-2 slides were stained with anti-TIF1β mouse monoclonal antibodies (a), anti-TIF1β antibody positive human autoimmune sera (b-e), or normal human serum (f). Serum dilution 1: 80; anti-TIF1β mAb, 1 μg/ml. ELISA, enzyme-linked immunosorbent assay; NHS, normal human serum; TIF, transcription intermediary factor.

The identity of the 120 kD protein immunoprecipitated by human sera as TIF1β was confirmed by IP-WB (Figure 2A). The proteins immunoprecipitated by human autoimmune sera were recognized by mAb to TIF1β, confirming the identity of the protein.

Antigen-capture ELISA using mouse anti TIF1β mAb was also performed using serially diluted anti-TIF1β positive sera (Figure 2B). All four anti-TIF1β positive sera were positive in ELISA in titers up to 1:12,500 to 1: 312,500. Control sera including anti-TIF1γ or anti-Mi-2 positive sera and NHS, were all negative.

### Western blot using affinity purified TIF1β

Strips of nitrocellulose filter with TIF1β affinity-purified by mAb were probed with mouse mAb anti-TIF1β and human autoimmune sera. Mouse mAb strongly reacted with the purified TIF1β protein; however, human autoimmune sera were negative when the western blot was developed using SuperSignal West Pico. Only the serum from case one was very weakly positive when developed using more sensitive SuperSignal West Femto (Figure 2C).

### Immunofluorescence staining of anti-TIF1β positive sera

HEp-2 ANA slides were stained with mouse anti-TIF1β mAb or human sera (Figure 2D). Mouse mAb showed a fine nuclear speckled pattern sparing the nucleoli and without chromosomal staining (panel a). Relatively monospecific serum from a Japanese patient (case one) showed a pattern very similar to that of mAb (panel b). Other sera (cases two to four) also showed a fine speckled nuclear staining pattern (panels c-e); however, one serum (case three) also had large nuclear speckles (panel d). A serum with coexisting anti-Mi-2 (case four) also had fine speckled nuclear staining (panel e).

### Clinical manifestations of patients with anti-TIF1β antibodies

Clinical features of anti-TIF1β antibody positive cases are summarized in Table 1. All four were women, three with a diagnosis of DM and one classified as undifferentiated connective tissue disease (UCTD) with elevated creatine phosphokinase (CPK) but without significant muscle symptoms. Thus, anti-TIF1β was found in 3/130 DM (1/35 Americans, 1/23 Japanese, 1/56 Mexicans) but 0/92 PM and none in SLE, SSc, or other conditions, suggesting that this specificity may be closely associated with DM, similar to antibodies to p155/140 (TIF1γ/α). The Japanese DM patient (case one) had elevated CPK (654 IU/L), mild muscle weakness, myalgia, a positive muscle biopsy and electromyogram (EMG); however, her myopathy resolved without treatment. The UCTD case (case two) also had elevated muscle enzymes, but muscle symptoms were not clear. Moreover, she had leukopenia, lymphopenia and autoimmune hemolytic anemia. This case also had a history of colon cancer (at age 41), cervical squamous metaplasia and fibroid tumors of the uterus. Two American patients (cases two and three) had mildly elevated levels of CPK (approximately 300 IU/L) that were controlled with low to moderate doses of steroids. The Mexican case also had high CPK but responded well to initial treatment.

## Discussion

In the present study, a 120 kD protein recognized by sera from four patients with DM or UCTD was identified as TIF1β based on mass spectrometric analysis and immunological confirmation, and it is closely related to a known cancer-associated DM autoantigen p155/140 (TIF1γ/α). Since the mobility of TIF1β in SDS-PAGE is identical to that of PL-12 (Figure 1), approximately 120 kD protein bands seen in IP will need to be cautiously interpreted. However, TIF1β and PL-12 have distinctive immunofluorescence patterns, which should help to differentiate the specificities. Anti-TIF1β produces a fine nuclear speckled pattern (Figure 2D) while anti-PL-12 gives a cytoplasmic pattern [24].

TIF1α, TIF1β, and TIF1γ belong to the TIF family of transcription cofactors and are part of a tripartite motif superfamily (TRIM24, 28, and 33, respectively) [23]. TIF1β/TRIM28, also known as Kruppel-associated box (KRAB)-associated protein 1 (KAP1), is a multi-functional protein involved in gene silencing, cell growth and differentiation, pluripotency, neoplastic transformation, apoptosis, DNA repair and the maintenance of genomic integrity [25]. A striking association of anti-p155/140 with cancer-associated DM has been described in several reports from the US [5], UK [7], Spain [9], Japan [6,8,10,11], and Korea [26] (reviewed in [9]), however, their association does not seem to apply to children [7] or young adults [11]. A Japanese PM/DM study that has just been published identified p140 as TIF1α and confirmed the p155 as TIF1γ [11]. In addition, seven cases with anti-TIF1β, four with anti-TIF1α and γ and two with anti-TIF1γ have been reported. It should be noted that in contrast to a previous study limited to PM/DM [11], anti-TIF1β antibodies were screened in 2,356 patients with various diagnoses in the present study, yet it was primarily associated with DM. Both studies suggest that anti-TIF1β is much less prevalent than anti-p155/140. Six of their 77 anti-p155/140 positive sera were also positive for anti-TIF1β [11] whereas none of our 23 anti-p155/140 were anti-TIF1β positive (data not shown). Although the difference observed between the two studies is not statistically significant, it is possible that coexisting patterns of autoantibodies to TIF family proteins are affected by genetic and/or environmental factors, selection bias (dermatology versus rheumatology clinic, for example) and other factors.

It has been shown that these TIF1 family proteins interact and work synergistically to suppress the development of malignancy in human and mouse models [23,27]. TIF1β is overexpressed in various types of cancer tissues [28] and is associated with progression or metastasis of the cancer [29,30]. It would not be surprising if the production of anti-TIF1β is also linked with cancer-associated DM as overexpression and modification of self-protein may trigger an autoimmune response. In the present study, one case with anti-TIF1β had a history of colon cancer, squamous metaplasia in a cervical smear and fibroid tumors of the uterus, but malignancy was not recorded in the other three cases, possibly due in part to a short follow-up period. A recent study [11] showed two of seven cases with anti-TIF1β had malignancy, possibly less frequently than anti-p155/140 positive cases.

In the case of the classic tumor suppressor gene p53, mutation and accumulation of p53 leads to the development of cancer as well as autoantibodies to p53, which is considered a result of broken immunological tolerance due in part to mutated p53 [31]. As TIF family proteins are known tumor suppressors [23,27] and are overexpressed in certain cancer tissues [28-30], a possible relationship between the development of autoantibodies to TIF and cancer may be explained by a similar mechanism. In this scenario, a mutation of TIF occurs as a primary event and triggers an autoimmune response against TIF, while cancer would develop due to the failed tumor suppressive activity of TIF. Further studies are necessary to determine whether anti-TIF1β has a strong association with cancer similar to anti-TIF-γ and whether TIF mutations are the primary event.

Alternative or additional mechanisms of anti-TIF1β production may be hypothesized based on the role of TIF1β in viral infections. TIF1β/KAP1 has been shown to play a critical role in controlling replication of Epstein-Barr virus [32], expression of murine endogenous retroviruses [33,34], latency regulation of Kaposi's sarcoma-associated herpesvirus (KSHV) [35], and replication of human papillomaviruses [36]. In this scenario, TIF1β would interact with viral proteins and the putative viral-self protein complex may trigger autoantibodies to TIF1β, similar to the induction of anti-p53 by a complex of viral simian virus 40 T protein and self p53 protein [37]. Association of virus and myositis autoantibody production has been suggested to occur by various pathways, including viral RNA interaction with aminoacyl tRNA synthetase and molecular mimicry of viral protein and autoantigens [38,39]. More recently, a target antigen of anti-CADM140 was identified as an intracellular viral RNA receptor, melanoma differentiation-associated gene 5 (MDA5) [13]. Thus, any of these mechanisms may also be involved.

## Conclusions

In summary, anti-TIF1β has been characterized as autoantibodies associated with DM with a mild form of myopathy without lung involvement. TIF1β is a multifunctional protein closely related to the known cancer-associated DM autoantigen p155/140 (TIF1γ/α) involved in suppression of malignancy as well as viral replication [23,27]. Both mutation of TIF1β and its interaction with viruses make an attractive hypothesis for the mechanism of production of autoantibodies to TIF1β. Future studies should verify an association of anti-TIF1β with malignancy and clarify mechanisms of its production.

## Abbreviations

ACR: American College of Rheumatology; ANA: antinuclear antibody; CADM: clinically amyopathic dermatomyositis; CPK: creatine phosphokinase; DM: dermatomyositis; ELISA: enzyme-linked immunosorbent assay; EMG: electromyography; IgG: immunoglobulin G; IIF: indirect immunofluorescence; HRP: horseradish peroxidase; ILD: interstitial lung disease; IP: immunoprecipitation; IP-WB: immunoprecipitation followed by western blot; KAP1: Kruppel-associated box (KRAB)-associated protein 1; KSHV: Kaposi's sarcoma-associated herpesvirus; mAb: monoclonal antibody; MDA5: melanoma differentiation associated antigen 5; MSA: myositis-specific autoantibodies; OD: optical density; PAPS: primary anti-phospholipid syndrome; PM: polymyositis; RA: rheumatoid arthritis; SAE: small-ubiquitin-like modifier-1 activating enzyme; SD: standard deviation; SLE: systemic lupus erythematosus; SSc: scleroderma; TIF: transcription intermediary factor; TRIM: tripartite motif; UCTD: undifferentiated connective tissue disease.

## Competing interests

The authors declare that they have no competing interests.

## Authors' contributions

MS, JYFC, SJR, YL, and YY carried out the immunoassays. MS and EKLC designed the study. MS performed the statistical analysis. YY, HY, MVM, MP, LJJ, MAS, CCR, ESS, WHR, and AC enrolled patients for the study, collected information and maintained databases. MS, AC, and EKLC drafted the manuscript. All authors read and approved the final manuscript.
